# Lifespan effects of current age and of age at the time of remembered events on the affective tone of life narrative memories: Early adolescence and older age are more negative

**DOI:** 10.3758/s13421-023-01401-x

**Published:** 2023-02-22

**Authors:** Theresa Martin, Nina F. Kemper, Florian Schmiedek, Tilmann Habermas

**Affiliations:** 1 Department of Psychology, Theodor-W.-Adorno-Platz 6, Goethe-Universität Frankfurt, D-60323 Frankfurt, Germany; 2 DIPF | Leibniz Institute for Research and Information in Education, Frankfurt, Germany; 3 International Psychoanalytic University Berlin, Berlin, Germany

**Keywords:** Life story, Lifespan, Autobiographical memory, Positivity effect, Positivity bump

## Abstract

The affective tone of autobiographical memories may be influenced by age in two ways—by the current age of the remembering individual and by the age of the remembered self at the time of the remembered event. While aging has been associated with more positive autobiographical memories, young adulthood is remembered more positively than other parts of life. We tested whether these effects also show in life story memories and how they act jointly on affective tone; also, we wanted to explore their effects on remembered lifetimes other than early adulthood. We tested effects of current age and age at event on affective tone with brief entire life narratives provided up to five times across 16 years by 172 Germans of both genders, ages 8 to 81 years. Multilevel analyses found an unexpected negativity effect of aging for current age and confirmed a “golden 20s” effect of remembered age. In addition, women told more negative life stories, and affective tone dipped in early adolescence for current age and was remembered as such up to mid-adulthood. Thus, the affective tone of life story memories is jointly influenced by current and remembered age. The absence of a positivity effect in aging is explained by the specific requirements of telling an entire life. We suggest the turmoil of puberty as a reason for the early adolescence dip. Gender differences are potentially explained by differences in narrative style, in depression rates, and in real-life challenges.

**Supplementary Information:**

The online version contains supplementary material available at 10.3758/s13421-023-01401-x.

Lives are different: Some lives are happier than others, depending on economic, social, and cultural factors, on chance, as well as on individual dispositions and on abilities and intentions to lead a good life. However, in most lives there are good and bad times. We are interested specifically in how the ups and downs of life are remembered, and whether there are systematic developmental or other age-related influences. Such influences may operate retrospectively when they are remembered (the rememberer’s *current age*) as well as at the time when life events had originally been experienced (*age at event*). To date, these influences have been studied separately. For rememberers’ current age, more positive evaluations of objects of attention and also of memories have been found in older age, the *positivity effect* (PE) of aging (Mather & Carstensen, 2005). For the age at the time when the remembered event happened, a peak of positive memories from early adulthood was found (Berntsen & Rubin, 2002), which we term *positivity bump* (in parallel to the *reminiscence bump* in the frequency distribution of memories). Developmental processes in old age and emerging adulthood have been adduced to explain these effects. We will show that to date, the quality of evidence for both the PE and the positivity bump in *autobiographical memories* (AMs) is strong for some forms of AMs, but weaker for others. Moreover, childhood and adolescence have been largely neglected but are an integral part of a lifespan perspective (Baltes et al., 1999). This paper studies simultaneously both temporal perspectives (cf. Bluck & Habermas, 2001), taking a lifespan developmental perspective on how remembering changes while life is being lived forward, and a life perspective on remembering, that is on the temporal distribution of memories across the past lifetime. We focus on both perspectives in an understudied form of the remembered life—life story memories. We first review the evidence for the PE in AMs, followed by evidence for the positivity bump in AMs, then consider possible constellations of the joint influence of current age and of age at the time of the remembered event, before testing the effects in a longitudinal study of brief life narratives covering a wide age range.

## Effects of current age on affective tone of autobiographical memories across the lifespan: The positivity effect of aging

When older relative to younger adults direct their attention (Charles et al., 2003; Mather & Carstensen, 2003) and when they recall and recognize material they learned in the lab (Mather & Carstensen, 2003; Petrican et al., 2008; Thomas & Hasher, 2006), their performance reflects a bias for positive items resulting either from a preference for positive over negative material or a lessened negativity bias typical of younger age (Carstensen & DeLiema, 2018). One possible explanation of the PE is that the age-related decline in cognitive processing capacities motivates older adults to prefer positive material which is easier to process (Labouvie-Vief, 2003). Alternatively, the PE has been explained by the motivation to pursue more emotionally meaningful goals with age due to the perceived dwindling of remaining time in life (Carstensen et al., 2003; Carstensen & Mikels, 2005; Mather & Carstensen, 2005). We first review studies with adult populations and then with children and adolescents.

The PE has been reported also for autobiographical memory (AM; e.g., Reed & Carstensen, 2012), based on evidence of retrospective judgements of self, for example, of one’s past health status (Kennedy et al., 2004). However, AMs are commonly defined as voluntary and explicit memories of episodes that took place in a spatial setting that was experienced by the remembering person (Rubin, 2019, 2022). The following review focuses on studies of age effects on voluntarily retrieved memories of self-experienced episodes and extended parts of one’s life.

Because findings of a PE in AMs are somewhat mixed, we distinguish three methods to elicit AMs that vary in the extent to which the selection of AMs is guided by criteria of self-relevance and of representing the entire life. These methodological differences reflect different everyday situations and functions of autobiographical remembering, ranging from stimulus-driven to highly self-selected and identity-driven uses (Bluck & Habermas, 2000; Koppel & Berntsen, 2015). Although entire life narratives, as studied here, occur relatively infrequently in everyday life, the life story functions as the necessary frame for any kind of autobiographical reasoning about the significance of past experiences for the self, and therefore for any kind of biographical self-presentation (Habermas & Bluck, 2000). When reporting studies for each of the three elicitation methods consecutively, we also specify whether the valence of or emotions associated with memories (termed *affective tone*) was assessed by participants or by external judges, without holding expectations for specific effects, and relate findings to the age composition of samples, expecting wider age ranges to produce larger age effects (cf. Reed et al., 2014).

When participants’ AMs are cued by positive, negative, and neutral words or by the affective tone of the event (termed *cued AMs*), memories do not need to be selected for self-relevance, but cues activate associative networks with episodic details (Habermas et al., 2015; Koppel & Berntsen, 2015). In such studies, the affective tone of AMs was typically rated by participants themselves, and comparisons were only between younger and older, but not middle-aged adults. Most of these studies provided evidence for the PE, that is, older compared with younger adults reported a greater/lower number of positive/negative memories (Barber et al., 2020; Comblain et al., 2005; Dijkstra & Kaup, 2005; Fernandes et al., 2008, Session 1; Ros & Latorre, 2010; Tomaszczyk & Fernandes, 2012), rated their AMs as more positive (Gallo et al., 2011; Schryer & Ross, 2012; no difference: Barber et al., 2020), and felt more positive about the event both at present (Ford et al., 2016; Tomaszczyk & Fernandes, 2012) and at the time of the event (Ford et al., 2016).

Other prompts activate self-relevance as a selection criterion by asking for self-defining AMs, AMs of turning points, low or high points, or for the most important, the happiest or saddest events compared with all other life events (termed *self-relevant AMs*). In these studies, affective tone was either self- or other-rated. Most of the studies in which participants themselves rated the affective tone of self-relevant AMs supported the PE comparing older to younger adults (Ikier & Duman, 2020; Quackenbush & Barnett, 2001; Rice & Pasupathi, 2010; Singer et al., 2007). One study did not support the PE (Siedlecki et al., 2015), possibly influenced by the relative old age of the youngest of three age groups (mean age 31 years). Of the only two studies in which the affective tone of self-relevant AMs was rated by researchers, the study with the wider age-range (18 to 95) confirmed the PE especially for the 70s and 80s (Webster & Gould, 2007), whereas the other study found fewer positive seven most important life memories in older than in younger adults (Bohn, 2010).

Finally, the elicitation method that requires the most self-relevance and additionally chronologically ordered events covering the entire lifetime refers to the life story by asking to divide the entire life into chapters, to draw the ups and downs of life as a line, or to narrate an entire life (termed *life story AMs*). The few studies of life story AMs showed the weakest support for the PE. Self-rated affective tone of life story AMs (life chapters) evidenced no PE when comparing younger, middle-aged, and older adults (Jensen et al., 2020; Thomsen et al., 2017). Life-lines of middle-aged and older men were more positive than those of younger men, whereas women exhibited the converse age distribution, suggesting a gendered PE (Assink & Schroots, 2002); in follow-up measurements 2 and 5 years later, age differences in affective tone were apparently no longer found (Assink & Schroots, 2010). To date, differences between younger and older adults in the affective tone of entire life narratives have not been studied. The study by Fromholt et al. (2003) only compared 80- with 100-year-olds’ externally rated affective tone of entire life narratives, finding no difference in valence.

To summarize, the PE for older compared with younger adults clearly showed in studies of cued AMs and in most studies of self-relevant AMs when affective tone was self-rated. The two studies of externally rated affective tone of self-relevant AMs provided somewhat contradictory evidence. Finally, the three studies of self-rated affective tone of life story AMs showed the least support for the PE. In addition, the use of a wider age range seemed to favour the PE.

It appears that the PE requires not only the freedom to evaluate past events more positively, which is provided by self-ratings of affective tone, but that in addition it also requires large degrees of freedom to select positive over negative AMs. This inference is suggested by the findings that life story AMs tended not to reproduce the PE even when their affective tone was self-reported. However, this inference is based on very few studies of life story AMs, and none of them are based on entire life narratives.

In addition, it would be helpful to paint a more fine-grained and continuous picture of how affective tone of autobiographical remembering develops across the lifespan by filling the gap between young and old adults left by most studies and by adding younger individuals. Findings on affective tone of AMs in childhood and adolescence are rare and unsystematic. Bauer et al. (2016) found 13-year-olds to rate their freely generated written AMs from ages 1–5 and 6–10 more positively than 20½-year-olds. Two studies analyzed affective tone of memories included in written entire life narratives, one finding no differences in the percentage of negative events between 10-, 12-, 13- and 14½-year-olds (Bohn & Berntsen, 2013), the second study finding a more negative affective tone of life narratives in 13- and 14½-year-olds’ than in 10½-year-olds (Ramsgaard & Bohn, 2021). We will explore age effects on affective tone of AMs in children and adolescents without formulating a hypothesis.

Taken together, there is a lack of studies of life story AMs, of longitudinal studies, and of studies from a lifespan perspective. To fill this gap, the present study of age-related effects on the affective tone of AMs is based on entire life narratives from a lifespan sample studied longitudinally.

## Effects of age at the time of the remembered events on affective tone of autobiographical memories across the remembered lifespan: The positivity bump

Extensive research demonstrates that a disproportionate number of early adulthood AMs is remembered by adults from age 40 onwards because only then the reminiscence bump differentiates itself from the higher number of memories from most recent times (recency effect; Rubin, 1986; Rubin et al., 1986; Rubin & Schulkind, 1997a). Memories from the bump have been found to be more positive than memories from other parts of life (Berntsen & Rubin, 2002). We review the distribution of affective tone of AMs across the entire remembered life, including studies of the positivity bump as well as the affective tone of other remembered life phases, which in most studies can be inferred from figures displaying the distribution of positive and negative memories separately.

Using word-cued AMs which were self-rated for affective tone, the positivity bump was confirmed by some studies (Alea et al., 2014; Janssen et al., 2011; Wolf & Zimprich, 2020; Zimprich & Wolf, 2018), but not by others (Janssen & Murre, 2008). Using self-relevant AMs, the positivity bump was confirmed by most studies, both if most positive and most negative memories were elicited separately (Berntsen & Rubin, 2002; Berntsen et al., 2011; Rubin & Berntsen, 2003; Thomsen et al., 2011; Zaragoza Scherman et al., 2015; except for Dickson et al., 2011, Study 2; Haque & Hasking, 2010, Study 1; Rubin & Schulkind, 1997b) and if most important memories were elicited first and then rated for affective tone by judges (Bohn, 2010) or by participants themselves (Glück & Bluck, 2007; Rubin & Berntsen, 2003; Zaragoza Scherman et al., 2017). Finally, also life story AMs self-rated on affective tone were most positive in the bump years of early adulthood (Assink & Schroots, 2002; Thomsen et al., 2014). Also, the only study including adolescents tended to show an increase in self-rated affective tone with age at event for both 13- and 20½-year-olds (Bauer et al., 2016).

These studies mostly included participants over age 40. In most of the studies that also included younger participants, a more positively remembered period in the 20s was apparent (Assink & Schroots, 2010; Berntsen & Rubin, 2002; Janssen & Murre, 2008; Thomsen et al., 2014; except Alea et al., 2014; Janssen et al., 2011), although there was no bump because the later drop in positivity was missing due to the too young ages of participants.

Taken together, irrespective of the varied elicitation methods, these studies point to a positivity bump in early adulthood compared with AMs from childhood and mid-adulthood. In most studies, however, the remembered lifespan was divided into 5- or 10-year age bins, which makes it difficult to describe the distribution of affective tone accurately across the remembered lives. Moreover, little is known how affective tone of children’s and adolescents’ AMs is distributed.

## Joint influence of current age and age at the time of the remembered events on affective tone of autobiographical memories

So far, influences of the two age parameters on the affective tone of AMs have been studied separately, raising the question whether and how they might interact with each other. We suggest four possible constellations. First, there may be a sole influence of current age on affective tone of AMs, such as more positive AMs in older age. Second, there may be an exclusive effect of age at event, such as young adulthood is remembered more positively. This possibility includes a spurious effect of current age, which depends on the precondition for effects of age at event to show that participants need to have reached that age, which is exemplified by the positivity bump demonstrating a clear downward swing only sometime in the fourth decade of life. Third, both current age and age at event may influence the distribution of affective tone independently, such as memories from all parts of life including the positivity bump in early adulthood becoming more positive in late adulthood due to the PE of aging. Fourth, current age and age at event might interact such that one moderates the influence of the other. For instance, only certain remembered parts of life might become more positive with aging; a nostalgia effect (or fading affect bias) would suggest that with age the positivity specifically of earlier years of life increases. To our knowledge, no study has yet analyzed these two age influences jointly so that we do not formulate hypotheses but will explore effects.

## The present study

Current age effects on the affective tone of AMs have been shown for the PE in studies of cued and self-relevant AMs, but it has rarely been studied in the life story. The three young–old comparisons of life story AMs did not find a PE, but none of them used the most encompassing representation of the life story—that is, entire life narratives. Also, only one of the studies (Assink & Schroots, 2010) was longitudinal, but did not report longitudinal findings for overall affective tone, which would have allowed to differentiate effects of aging from cohort effects, derived, for instance, from experiences of historical events such as WWII (e.g., Bohn, 2010). Furthermore, the development of the affective tone of children and adolescents is so far underrepresented in the literature. Studies of the distribution of affective tone by age at event have focused on the positivity bump but lack analyses of the remaining parts of remembered life as well as the use of life story AMs and of age-diverse samples. Finally, there is a lack of joint analyses of both age effects for which entire life narratives offer a unique advantage because they cover the entire remembered life of individuals of different ages that allow both within- and between-person comparisons. To fill these gaps in the literature, the present study of age-related effects on the affective tone of AMs is based on entire life narratives from a lifespan sample studied longitudinally.

First, based on the PE of aging we hypothesized that with increasing age, the overall affective tone of life narratives becomes more positive (H1). Moreover, we aimed at exploring the development of overall affective tone of life narratives in childhood and adolescence. Second, we intended to analyze the effects of age at the time of the remembered event on the affective tone of life narrative memories irrespective of current age, expecting to confirm the positivity bump of early adulthood (H2). Third, we wanted to explore the joint influence of both age parameters. Finally, we wanted to explore similarities and differences between the distribution of affective tone of entire life narratives across the lifespan (participants’ current age) and the distribution of affective tone of memories across the remembered lifespan (age at event).

## Methods

We present secondary analyses of data collected in the longitudinal MainLife project, which originally had been established to examine the development of the ability to construct a coherent life narrative (for a list of all publications from MainLife, see Habermas, 2022). Thus, we did not determine the sample size a priori but report the final sample, all data exclusions, all manipulations, and all measures included in the study.

## Participants

MainLife is a longitudinal lifespan study of brief entire life narratives with 4-year intervals. The present study comprises all five waves collected in 2003, 2007, 2011, 2015, and 2019. In 2003, we started with four younger cohorts roughly aged 8, 12, 16, and 20 (*N* = 114). In 2007, two adult cohorts were added, roughly aged 40 and 65 (*N* = 58). Dropout rates for younger cohorts were fairly low in 2007 (*N* = 104; −8.8%) and 2011 (*N* = 99; −4.8%), a little higher in 2015 (*N* = 87; −12.1%), and the number of participants increased in 2019 (*N* = 90; +3.4%). Dropout rates were a little higher for the older two cohorts in 2011 (*N* = 51; −12.1 %), but low in 2015 (*N* = 48; −5.9%) and 2019 (*N* = 47; −2.1%). Some participants missing in one wave participated again in a later wave. Overall affective tone of life narratives did not differ significantly between participants who participated versus dropped out four years later for younger and older cohorts (*p*s > .05; Table S1). Gender was roughly equally distributed across cohorts and measurement occasions (Table 1). Additional control participants (*N* = 28) who were only interviewed in 2019 were not included in this study.

**Table 1:** Age of participants in years (mean, standard deviation, age range), number of participants, and gender distribution

	Cohort 1	Cohort 2	Cohort 3	Cohort 4	Cohort 5	Cohort 6	*n*
Wave 1	8.58 (0.27)	12.41 (0.37)	16.53 (0.44)	20.50 (0.57)			
Range	8.05–9.13	11.87–13.22	15.84–17.58	19.32–21.48			
* n*	27	31	28	28			114
Females	13	17	13	15			
Wave 2	12.90 (0.52)	16.58 (0.42)	20.70 (0.51)	24.93 (0.73)	41.39 (2.86)	65.38 (2.73)	
Range	11.82–13.73	15.92–17.41	19.73–21.75	23.09–26.21	35.10–46.41	59.41–69.48	
* n*	23	29	26	26	28	30	162
Females	12	16	12	14	14	15	
Wave 3	17.03 (0.48)	20.58 (0.39)	24.61 (0.41)	28.90 (0.67)	45.08 (3.02)	68.77 (2.58)	
Range	15.83–17.69	19.84–21.39	23.84–25.73	27.19–29.73	39.32–49.74	63.66–72.71	
* n*	23	27	26	23	22	29	150
Females	13	15	11	13	11	15	
Wave 4	21.31 (0.61)	25.07 (0.43)	29.20 (0.65)	33.49 (0.77)	49.44 (2.89)	73.33 (2.56)	
Range	20.01–22.59	24.46–26.28	28.09–30.77	31.59–34.49	42.88–52.75	68.05–77.11	
* n*	24	22	20	21	20	28	135
Females	13	13	11	11	9	14	
Wave 5	25.32 (0.51)	28.87 (0.34)	33.03 (0.68)	37.09 (0.77)	53.83 (3.03)	77.02 (2.57)	
Range	24.08–26.25	28.17–29.67	31.83–34.75	35.33–38.08	47.17–58.33	71.92–80.58	
* n*	23	23	22	22	20	27	137
Females	12	14	10	13	9	14	
Total							698

Cohorts 2, 3, and 4 were recruited from among (former) students of a German Gymnasium, a German higher-track high school. The youngest participants (Cohort 1) were recruited from the higher performing half of third graders in one elementary school to make their level of academic achievement comparable to the older participants. The two older cohorts were recruited in the university neighbourhood via flyers and among continuing education university students. In 2019, educational level was high: 88.4 % with the highest school degree (Abitur or Fachabitur), 9.4% with an intermediate school degree (Mittlere Reife), 2.2% with the lowest (Hauptschulabschluss). Most participants were married (31.6%) or single (30.1%), 24.3% in a relationship, 11.0% divorced/separated, 2.9% widowed. Almost half of the younger participants had at least one parent who was born outside Germany (47.6%), which was less frequent in the two older cohorts (19.6%), reflecting historical developments in the local population. All participants were fluent in German. Participation was compensated with 20 Euros in 2003, 40 Euros in 2007 and 2011, and 60 Euros in 2015 and 2019.

## Procedure

Life narratives were collected by different female interviewers in each wave. In 2003, we collected two life narratives two weeks apart (cf. Habermas & de Silveira, 2008), of which we only included the first in the present study. In Wave 1, participants were interviewed in their schools, and in Waves 2 to 5 in the lab, and few at their homes. After giving informed consent (which had also been collected from parents for minors beforehand), participants were instructed to write down their seven most important memories on index cards and to date them. They ordered the cards chronologically on the table in front of them. Then, participants freely narrated their life story in about 15 minutes, including the seven memories and showing how they had become the person they are today (for instructions cf. Habermas & de Silveira, 2008). Then they filled in several questionnaires that are not part of this study. This resulted in up to five life narratives for the younger and up to four life narratives for the older cohorts, giving a total of *N* = 698 life narratives from a total of 172 participants that were included in the analyses.

## Measures

In this longitudinal study, often the same text segmenting, rating, or coding procedure was done for (several) consecutive waves by different pairs of coders/raters. To ensure stability in the application of manuals, coders/raters were always trained with the material prepared by their predecessors.

### Transcription of life narratives and division into propositions

Life narratives were audio-recorded, transcribed verbatim, and divided into propositions. For each wave, two coders independently divided 40 transcripts into main or subordinate clauses (agreement for propositioning between 96.0% and 98.6%). Half of each set of remaining life narratives was divided into propositions by one of the respective two coders.

### Segmentation of life narratives

Segments consist of at least four thematically related propositions. Prototypically they regard a specific event but may also cover extended events or evaluative summaries. If segment borders deviated by up to one proposition, this counted as agreement. Segmentation was done for Waves 1 and 2, for Wave 3, and again for Waves 4 and 5 by two coders each. After reaching agreement between two coders for 32 life narratives (Cohen’s κ = .80 both for Waves 1 and 2 as well as for Wave 3, κ = .83 for Waves 4 and 5), each coder independently divided half of the remaining transcripts into segments. Additional follow-up interrater reliabilities based on 16 randomly selected life narratives were calculated during the segmentation process to ensure continued reliability (κ = .81, κ = .92, and κ = .89). Discrepancies were discussed and thereby resolved. In the following, we address segments also as *memories*.

### Affective tone of segments and overall affective tone of life narratives

Each segment was coded for *affective tone* using six codes, based, if available, on participants’ explicit evaluation. Here we only use positive (+1), neutral (0), ambivalent (0), and negative (−1) codes. The remaining two codes contamination (a change from initially positive to negative—for example, friendship ending with a conflict) and redemption (a change from initially negative to positive, e.g., positive ending of an accident; McAdams et al., 2001) were not included in our analyses, because they indicate a change of valence which cannot be represented on the positive-negative dimension of valence. Affective tone of segments was coded for Waves 1 to 3 and again for Waves 4 and 5. Reliabilities showed high interrater agreement with κ = .76 (32 life narratives) and κ = .89 (16 life narratives for follow-up reliability) in Waves 1 to 3 as well as in Waves 4 and 5 with κ = .76 (32 life narratives), but slightly lower follow-up interrater agreement (κ = .60).

For the entire life narrative, we calculated the *overall affective tone* by averaging codes across all segments, excluding those coded as contamination or redemption, and multiplying the value by 100, resulting in values ranging between +100 (only positive) and −100 (only negative; cf. Camia & Habermas, 2020). For instance, if a participant’s life narrative comprised 15 positive, five neutral/ambivalent, and five negative segments, the overall affective tone of the life narrative was $\left(\frac{15\;\times\;\left(+1\right)\;+\;5\;\times\;(0)\;+\;5\;\times\;\left(-1\right)}{25}\right)\times100=40$.

### Age at the time of the remembered event

The *age at the time of the remembered event* (short: *age at event*) covered by each segment was estimated on the basis of references to age and dates (cf. Köber et al., 2015). For segments covering extended events, age at event was calculated as the mean of the age at the beginning and at the end of the segment (cf. Habermas & Diel, 2013). If two events were mentioned in one segment, the first was used to code age at event. We also used a list of specific times for colloquial terms for temporal distance (e.g., recently = 2 weeks ago; a few years ago = 2 years ago). General statements about life circumstances (e.g., describing family members) were coded as current age of participants. Because age at event was unambiguous, no interrater reliability was calculated (cf. Camia & Habermas, 2020; Köber et al., 2015).^1^ Events within a month of participants’ future were included as part of the extended present; roughly half a percent of segments (*n* = 71) regarded participants’ more distant future and were therefore excluded from further analyses.

### Content of memories

All segments of life narratives were attributed to one of 97 possible event categories, of which 68 were taken from Berntsen and Rubin (2004) and Habermas (2007). An additional 29 event categories, taken from the life narratives of the adult cohorts, were added to cover life story events of the entire lifespan. Event categories were coded for Waves 1 to 3 and again for Waves 4 and 5. Interrater agreement was high, κ = .84 (32 life narratives) and κ = .74 (16 life narratives for follow-up reliability) in Waves 1 to 3 and κ = .87/.69 in Waves 4 and 5.

## Data analyses

We used the lmer function of the lme4 package (Bates et al., 2015) with maximum-likelihood estimation for all tests of hypotheses and created figures with the ggplot2 package (Version 3.4.0; Wickham, 2016) in RStudio (Version 2022.12.0). To test the first hypothesis of an increase of the overall affective tone of life narratives with current age, we applied multilevel models to account for the nested data structure with multiple life narratives per participant. Younger (8–36 years) and older cohorts (40–81 years) were all analyzed in one model to investigate the current age-related effect. To identify the best fitting multilevel model, we first tested fixed and random intercepts. Second, we included gender as a control variable (cf. Assink & Schroots, 2002) before entering a fixed effect of current age of participants (scaled in decades to aid the estimation of quadratic and cubic effects and centered at 8 years [= 0.8 decades] to set the intercept at the youngest age of the cohorts). Then, a fixed effect of current age within-person centred at baseline (= participants’ individual youngest age at first measurement) was entered to test for a longitudinal increase with age. Resulting models were compared by testing for significant improvements in model fit using χ^2^ tests of the deviance statistic. Confidence intervals of parameters were estimated with bootstrapping.

To explore the second research question, we inspected the distribution of affective tone of segments across age at event. To test the second hypothesis of the positivity bump, including all participants and ignoring current age, we used multilevel models in which segments at Level 1 were nested in life narratives at Level 2 which in turn were nested in participants at Level 3. Affective tone of segments was predicted by age at event (baseline-centred to analyze the change from the first memory and divided by 10 to aid estimations) and was included as a linear and higher-order terms to model the trajectory across the remembered life.

To answer the third question about possible combined effects of age at event and current age, we explored the variation of affective tone by age at event separately for age groups (not cohorts) and compared the age distribution of overall affective tone of life narratives with the trajectories of affective tone of life narrative memories.

## Results

We first present descriptive statistics regarding overall affective tone and current age to then test Hypothesis 1 of an increase of affective tone with age. Next, we present descriptive statistics regarding affective tone and age at event to then test Hypothesis 2 of a positivity bump in memories, irrespective of current age. Finally, we explore the joint influence of both age parameters on affective tone and compare the course of overall affective tone of life narratives across the lifespan with the course of affective tone of events across the remembered lifespan for exploratory purposes.

## Effects of current age on overall affective tone of life narratives

### Descriptive characteristics

**Fig. 1:**
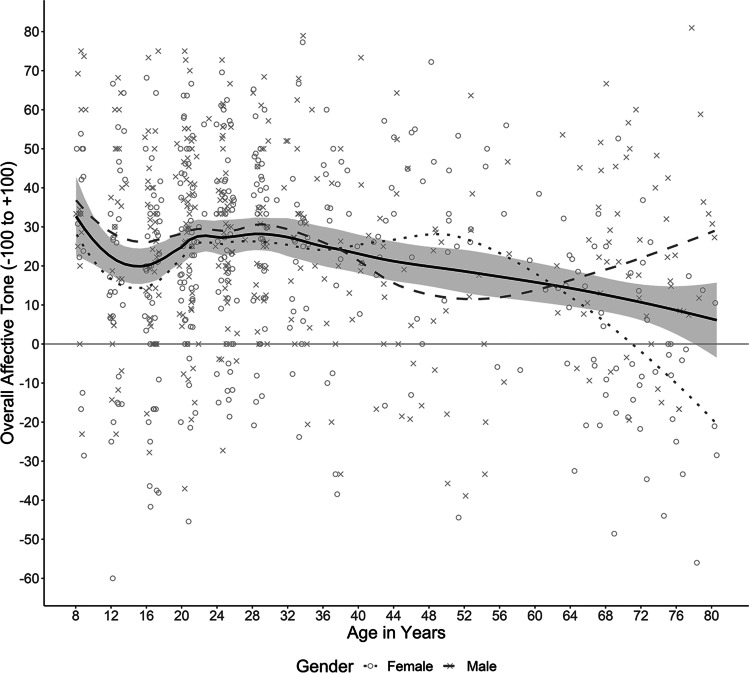
Mean overall affective tone of life narratives by participants’ current age. *Note*. Each point represents a life narrative (*N* = 698). Trend lines were fitted using the locally weighted polynomial regression (*loess*) method (α = .5, that is, each local regression to produce the trend lines included 50% of the total data points; cf. Jacoby, 2000) as implemented in the R package ggplot2 (Wickham, 2016). The solid trend line corresponds to the overall mean. The 95% confidence interval for the mean overall affective tone is displayed in grey

Across cohorts, with age life narratives became longer (number of segments; *r* = .31). In total, *N* = 698 life narratives at Level 1 were nested in 172 participants at Level 2. Across all life narratives, mean overall affective tone was positive (*M* = 22.39, *SD* = 25.54). To describe the relationship between overall affective tone of life narratives and participants’ current age, we plotted the two variables and included trend lines (Fig. 1).

**Fig. 2:**
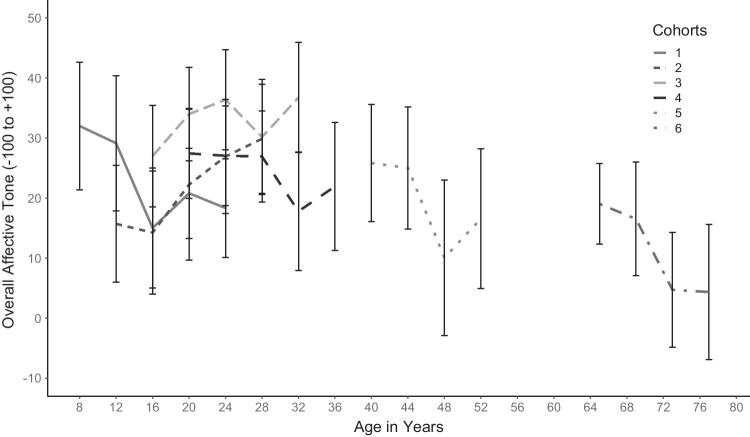
Longitudinal course across 12 (Cohorts 1 to 6) and 16 years (Cohorts 1 to 4) of mean overall affective tone of life narratives. *Note.* Error bars display the 95% confidence interval

Except for middle-aged adults, women narrated their lives more negatively. After the mid-20s, there was a constant decline in affective tone, while between childhood and the mid-20s overall affective tone showed a U-shaped course with a low between ages 12 to 16 (Table S2). This general age trend also showed in each cohort’s separate longitudinal trajectory, particularly a linear decrease in middle-aged and older adults with age (Fig. 2).

### Tests of Hypothesis 1

We had hypothesized that the overall affective tone of life narratives becomes more positive with age. To estimate how much of the total outcome variability in overall affective tone of life narratives (Level 1) can be attributed to narratives being narrated by the same participant (Level 2), we calculated an intraclass correlation (ICC). The resulting *ρ*_ICC_ = .42 indicates that almost half of the total variance can be attributed to between-person differences, necessitating the use of multilevel models.

We first tested for fixed and random intercepts and retained the better fitting model with the random intercept. Second, we consecutively added fixed effects of gender and of both current age (centred at 8 years and baseline-centred; Table 2). While there was no significant within-person longitudinal effect of age, overall affective tone was unexpectedly negatively predicted by current age (Model 3). Thus, contradicting the hypothesis, older participants had more negative life narratives.

**Table 2:** Comparison of multilevel models for the effect of current age on overall affective tone of life narratives

	Model 0		Model 1		Model 2		Model 3		Model 4		Model 5	
	Estimate (*SE*)	95% CI	Estimate (*SE*)	95% CI	Estimate (*SE*)	95% CI	Estimate (*SE*)	95% CI	Estimate (*SE*)	95% CI	Estimate (*SE*)	95% CI
Fixed Part												
Intercept	22.22* (1.48)	[19.28, 24.83]	18.85* (2.04)	[14.50, 23.16]	25.11* (2.59)	[20.09, 30.46]	24.95* (2.61)	[20.22, 30.07]	19.65* (3.14)	[13.26, 25.74]	20.38* (3.56)	[13.45, 27.61]
Male			6.89* (2.92)	[0.57, 12.37]	6.96* (2.82)	[0.89, 12.50]	7.00* (2.82)	[1.23, 12.49]	7.17* (2.82)	[1.61, 12.87]	7.17* (2.83)	[1.84, 13.09]
Age^a^					−2.40* (0.65)	[−3.55, −1.19]	−2.55* (0.70)	[−3.76, −1.16]	3.18 (1.94)	[−0.51, 7.04]	1.71 (3.89)	[−5.73, 9.42]
Age^b^							0.08 (0.15)	[−0.21, 0.33]				
Age^2^									−0.85* (0.28)	[−1.41, −0.31]	−0.25 (1.40)	[−2.84, 2.50]
Age^3^											−0.06 (0,14)	[−0.32, 0.19]
Random Variance												
Residual ${\upsigma}_e^2$	384.84 (19.62)	[18.34, 20.77]	384.51 (19.61)	[18.52, 20.87]	383.01 (19.57)	[18.31, 20.83]	382.88 (19.57)	[18.44, 20.70]	376.27 (19.40)	[18.24, 20.49]	375.86 (19.39)	[18.23, 20.60]
Intercept ${\tau}_1^2$	273.55 (16.54)	[14.24, 19.04]	262.72 (16.21)	[13.83, 18.36]	239.50 (15.48)	[12.87, 17.90]	239.41 (15.47)	[12.92, 17.50]	241.47 (15.54)	[12.94, 17.62]	242.35 (15.57)	[12.71, 17.69]
Model Fit												
Deviance	6364.42		6358.93		6345.41		6345.15		6336.15		6335.96	
Δχ^2^			5.49*		13.52*		0.26		9.27*		0.19	
*df*			1		1		1		1		1	

CI = confidence intervals (based on bootstrapping). *N* = 698 life narratives. Sequentially adding predictors was tested with Δ*χ*^2^ tests based on model deviance (−2Log-Likelihood), comparing it to the previous model (except Model 4 was compared with Model 2).

^a^Age was divided by 10 and centred at 0.8 decades (according to the youngest age group) to aid the estimation of quadratic and cubic effects.

^b^Age was within-person baseline-centred at the individually youngest age at the first measurement.

*p < .05, one-tailed

To model the unexpected nonlinear variation of overall affective tone with current age in Fig. 1 for exploratory purposes, we consecutively added the predictors age^2^ and age^3^. Besides gender and age, also age^2^ significantly predicted the overall affective tone (Model 4). Women narrated their lives more negatively compared with men, and overall affective tone was more negative especially for older participants. This model explained 11.73% variance on Level 2 between participants and 2.22% variance on Level 1 within participants.

### Separate exploration of younger and older cohorts

Because Fig. 1 shows a different curve for ages up to about 36 years and for later ages, we exploratorily conducted further separate analyses for younger versus middle-aged to older adults. Again, we first included gender, then current age (located at eight [= 0.8 decades] respectively 40 years [= four decades]). Guided by descriptive results, we specifically tested a quadratic and cubic effect of current age in Cohorts 1 to 4, as well as a linear longitudinal decrease (baseline-centred current age) and an Age × Gender interaction in Cohorts 5 and 6.

For the younger participants, besides a linear and quadratic also a cubic trend of overall affective tone proved significant (γ_Age_ = −29.81, 95% CI [−52.27, −9.96]; γ_Age_^2^ = 25.62, 95% CI [9.38, 43.45]; γ_Age_^3^ = −5.76, 95% CI [−9.69, −2.08], all *p*s < .05), reflecting the dip in early adolescence and a renewed slow decrease after age 24 (Fig. 1). Women’s life narratives were overall more negative than those of men (γ = 6.86, 95% CI [0.16, 13.40]). Adding interactions between gender and age did not increase model fit. The final model explained 5.59% variance on Level 2 between participants and 2.57% variance on Level 1 within participants.

For middle-aged and older participants, the best fitting model (AIC = 1834) included the significant predictors (all *p*s < .05) current age (γ = −6.86, 95% CI [−12.25, −1.64]), current age baseline-centred (γ = −1.07, 95% CI [−1.72, −0.31]), and the significant interaction Age × Gender (γ = 9.42, 95% CI [2.81, 15.57]), as well as the nonsignificant predictor gender (*p* > .05). This indicates that higher age was related to more negative affective tone of life narratives, that individual life narratives became more negative longitudinally, and older men narrated more positively than older women, whereas the opposite pattern was true for middle-aged participants. The final model explained 18.56% variance on Level 2 between participants and 13.48% variance on Level 1 within participants.

To explore possible factors explaining this age-related gender difference in affective tone, we examined the role of having children and relationship status, as these variables were not evenly distributed across age and gender. Adding children (childless vs. having children) to the linear multilevel model did not improve model fit (AIC = 1834) but adding relationship status (single/widowed/separated/divorced vs. in a relationship/married) did (AIC = 1830, ${\chi}_{df=1}^2$ = 5.94). Overall affective tone of life narratives of middle-aged and older adults was significantly predicted (all *p*s < .05) by both current age variables (centred at 4 decades: γ = −6.97, 95% CI [ −11.72; −1.57]; baseline-centred: γ = −0.94, 95% CI [−1.59; −0.30]), Age × Gender (γ = 9.68, 95% CI [2.17; 14.95]), as well as relationship status (γ = 8.34, 95% CI [1.70, 17.44]), but not gender (*p* > .05). Thus, middle-aged and older adults who were in a relationship had more positive life narratives compared with participants who were not, but the interaction between age and gender remained significant. This model explained an additional 7.14% of the variance on Level 2 between participants and 1.65% of the variance on Level 1 within participants compared with the previous model.

## Distribution of affective tone of memories across the remembered life

### Descriptive characteristics

**Fig. 3:**
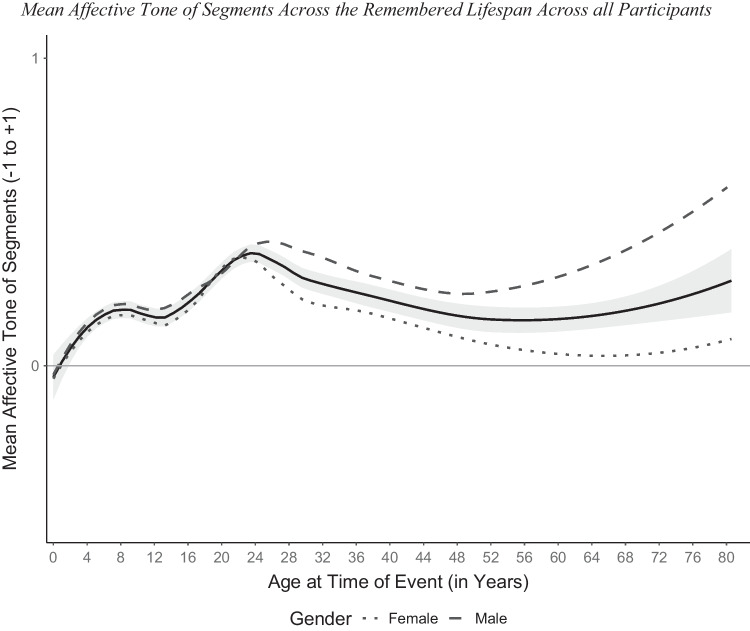
Mean affective tone of segments across the remembered lifespan across all participants. *Note.* Trend lines were included using the loess method (α = .5) as implemented in the R package ggplot2 (Wickham, 2016). The 95% confidence interval of the overall mean is displayed in grey

Turning from participants’ current age and entire life narratives to ages at the time of each remembered event, there were *N* = 15,353 segments nested in life narratives (*N* = 698) which were nested in participants (*N* = 172). Since we were only interested in pure valence and not change in valence and because the latter was rare (9.3%), we excluded segments coded for redemption (*n =* 910) and contamination (*n =* 522), resulting in *N* = 13,921 segments.^2^ Overall, there were *n* = 2,462 negative (17.69%), *n* = 5851 neutral or ambivalent (42.03%), and *n* = 5,608 positive (40.28%) segments. We examined the trajectory of mean affective tone of segments across the remembered life in the entire lifespan sample by plotting these two variables and inserting trend lines (Fig. 3).

Overall, memories up to age 8 increased in positivity followed by a slight decrease and relative dip between 12 and 16 years of age at the remembered event. A clear bump of more positive memories appeared across emerging adulthood (between 20 to 32 years) after which gender differences become evident. Across middle adulthood, mean affective tone remained relatively stable but increased again slightly for the highest ages at event (roughly from age 72), which was particularly true for older men who generally outperformed women in their positivity of memories.

### Tests of Hypothesis 2

To test the positivity bump of age at event, we applied multilevel models. The three-level model with segments (Level 1, *N* = 13921) nested in life narratives (Level 2, *N* = 698) nested in participants (Level 3, *N* = 172) had a better fit than the two-level model (nesting segments only in life narratives; Table 3). The ICC between life narratives on Level 2 was slightly higher (*ρ*_ICC_ = .09) than between participants on Level 3 (*ρ*_ICC_ = .06).

**Table 3:** Multilevel models analyzing the distribution of affective tone of segments across the remembered life for all participants

	Model 0 (2 levels)		Model 0 (3 levels)		Model 1		Model 2		Model 3	
	Estimate (*SE*)	95% CI	Estimate (*SE*)	95% CI	Estimate (*SE*)	95% CI	Estimate (*SE*)	95% CI	Estimate (*SE*)	95% CI
Fixed Part										
Intercept	0.24* (0.01)	[0.22, 0.26]	0.24* (0.02)	[0.20, 0.27]	0.20* (0.02)	[0.16, 0.24]	0.13* (0.02)	[0.08, 0.18]	0.09* (0.03)	[0.04, 0.14]
Male					0.07* (0.03)	[0.01, 0.13]	0.07* (0.03)	[<0.01, 0.14]	0.07* (0.03)	[0.01, 0.14]
Age at Event Age at Event^2^ Age at Event^3^ Age at Event^4^ Age at Event^5^							0.04* (<0.01)	[0.03, 0.05]	0.09* (0.01) −0.01* (<0.01)	[0.07, 0.11] [−0.01, −0.00]
Age										
× Age at Event × Age at Event^2^ × Age at Event^3^ × Age at Event^4^ × Age at Event^5^										
Random Variances										
Level 1 ${\upsigma}_e^2$	0.48 (0.69)	[0.22, 0.26]	0.48 (0.69)	[0.68, 0.70]	0.48 (0.69)	[0.68, 0.70]	0.47 (0.69)	[0.68, 0.70]	0.47 (0.69)	[0.68, 0.70]
Level 2 ${\tau}_2^2$	0.05 (0.22)	[0.21, 0.24]	0.02 (0.13)	[0.11, 0.15]	0.02 (0.13)	[0.11, 0.15]	0.02 (0.14)	[0.12, 0.15]	0.02 (0.14)	[0.12, 0.15]
Level 3 ${\tau}_3^2$			0.03 (0.18)	[0.15, 0.21]	0.03 (0.17)	[0.15, 0.20]	0.04 (0.19)	[0.16, 0.21]	0.03 (0.19)	[0.16, 0.21]
Model Fit										
Deviance	29955.65		29809.90		29805.21		29716.58		29696.32	
Δχ^2^			145.75*		4.69*		88.63*		20.27*	
*df*			1		1		1		1	

	Model 4		Model 5				Model 6		Model 7	
	Estimate (*SE*)	95% CI	Estimate (*SE*)	95% CI			Estimate (*SE*)	95% CI	Estimate (*SE*)	95% CI
Fixed Part										
Intercept	0.05 (0.03)	[−0.01, 0.10]	0.06* (0.03)	[0.01, 0.12]			0.08* (0.03)	[0.03, 0.14]	0.30* (0.04)	[0.22, 0.37]
Male	0.07* (0.03)	[0.01, 0.14]	0.07* (0.04)	[0.01, 0.14]			0.07* (0.03)	[0.01, 0.13]	0.07* (0.03)	[0.02, 0.13]
Age at Event Age at Event^2^ Age at Event^3^ Age at Event^4^ Age at Event^5^	0.18* (0.02) −0.05* (0.01) 0.004* (<0.01)	[0.14, 0.23] [−0.07, −0.03] [<0.01, 0.01]	0.12* (0.02) −0.00 (0.03) −0.01 (0.01) 0.00* (<0.01)	[0.04, 0.20] [−0.06, 0.05] [−0.02, <0.01] [−0.00, <0.01]			0.02 (0.06) 0.11* (0.06) −0.05* (0.02) 0.01* (<0.01) −0.00* (<0.01)	[−0.11, 0.14] [0.01, 0.24] [−0.10, −0.00] [0.00, 0.02] [−0.00, −0.00]	−0.30* (0.10) 0.40* (0.13) −0.19* (0.06) 0.04* (0.01) −0.002* (<0.01)	[−0.51, −0.07] [0.11, 0.65] [−0.30, −0.04] [<0.01, 0.06] [−0.00, −0.00]
Age									−0.12* (0.01)	[−0.15, −0.10]
× Age at Event × Age at Event^2^ × Age at Event^3^ × Age at Event^4^ × Age at Event^5^									0.12* (0.03) −0.06* (0.03) 0.02 (0.01) −0.003* (<0.01) <0.01* (<0.01)	[0.06, 0.18] [−0.12, −0.00] [−0.00, 0.04] [−0.01, 0.00] [0.00, 0.00]
Random Variances										
Level 1 ${\upsigma}_e^2$	0.47 (0.69)	[0.68, 0.70]	0.47 (0.69)	[0.68, 0.69]			0.47 (0.69)	[0.68, 0.69]	0.47 (0.68)	[0.68, 0.69]
Level 2 ${\tau}_2^2$	0.02 (0.13)	[0.12, 0.16]	0.02 (0.14)	[0.12, 0.16]			0.02 (0.13)	[0.11, 0.16]	0.02 (0.13)	[0.11, 0.15]
Level 3 ${\tau}_3^2$	0.03 (0.18)	[0.15, 0.21]	0.03 (0.18)	[0.15, 0.21]			0.03 (0.18)	[0.15, 0.21]	0.03 (0.16)	[0.13, 0.19]
Model Fit										
Deviance	29674.66		29671.38				29666.66		29516.38	
Δχ^2^	21.66*		3.28*				4.72*		150.29*	
*df*	1		1				1		6	

CI = confidence interval (based on bootstrapping). <0.01 = values between 0 and 0.01. Variances are between segments (${\upsigma}_e^2\Big),$ life narratives (${\tau}_2^2\Big),$ and participants (${\tau}_3^2\Big)$. Age at event was baseline-centred and divided by 10, and age was divided by 10 and centred at the youngest age group (0.8 decades) to aid the estimation of higher-order effects. Sequentially adding predictors was tested with Δ*χ*^2^ tests based on model deviance (−2Log-Likelihood).

*p < .05, one-tailed

First, we included gender before modelling the trajectory of affective tone of segments across the remembered lifespan by adding fixed effects of age at event consecutively (linear to fifth degree polynomial; baseline-centred and divided by 10). Affective tone became more positive with increasing age at event and was higher for men compared with women. It was distributed following a curvilinear trajectory (Model 3 in Table 3) peaking in the 20s across all participants (Fig. 3), thus, confirming a positivity bump in early adulthood. The curvilinear shape of the distribution was maintained when adding higher order effects of age at event for exploratory purposes (Models 4–6 in Table 4).

**Table 4:** Content of three most frequent positive and negative childhood memories (up to remembered age 8)

	Positive Memories (*n*)	Negative Memories (*n*)
Cohorts 1 to 4	Begin daycare (122)	Separation of parents (39)
	Go to school (89)	Family situation (30)
	Having peers (73)	Begin daycare (22)
	Total (701)	Total (242)
Cohort 5	Family situation (14)	Serious disease (13)
	Birth (8)	Not severe illness or accident (13)
	Activities with family member (6)	Family situation (7)
	Total (63)	Total (55)
Cohort 6	Family situation (9)	War (92)
	Go to school (7)	Family situation (26)
	Leisure activity (5)	Others' death (12)
	Total (48)	Total (194)

Total refers to the total number of positive/negative segments of childhood memories (until 8 years of age at event) in entire life narratives across all waves.

## Exploration of joint influence of current age and age at event on affective tone

The literature suggests that the shape of a positivity bump only occurs once a decrease in affective tone sometime in the 30s turns a high of the 20s into a bump. Although this age dependency of the positivity bump does not imply change in the distribution of remembered affective tone by current age, we wanted to explore whether the influence of age at event varied by current age or vice versa. Therefore, we added current age and its interactions with all age at event variables (linear to fifth degree polynomial) as additional predictors to the model testing joint influences on affective tone of segments (Model 7). In addition to the significant higher-order effects of age at event, again (like in Model 2, Table 2) older participants had more negative memories. Moreover, most cross-level interactions were significant, indicating that the trajectories of affective tone of life narrative memories change with current age. This model explained an additional 0.72% of variance between memories, 9.92% of variance between life narratives, and 20.35% of variance between participants compared with the model with only gender and all higher-order age at event (Model 6, Table 3).

**Fig. 4:**
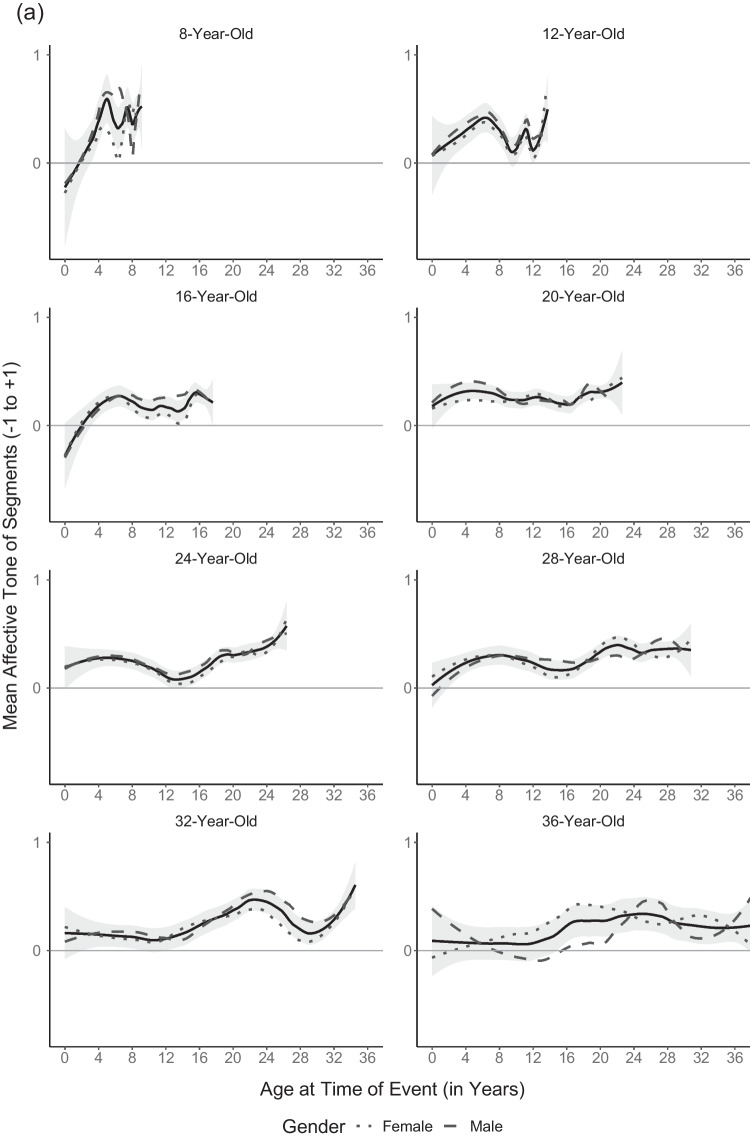

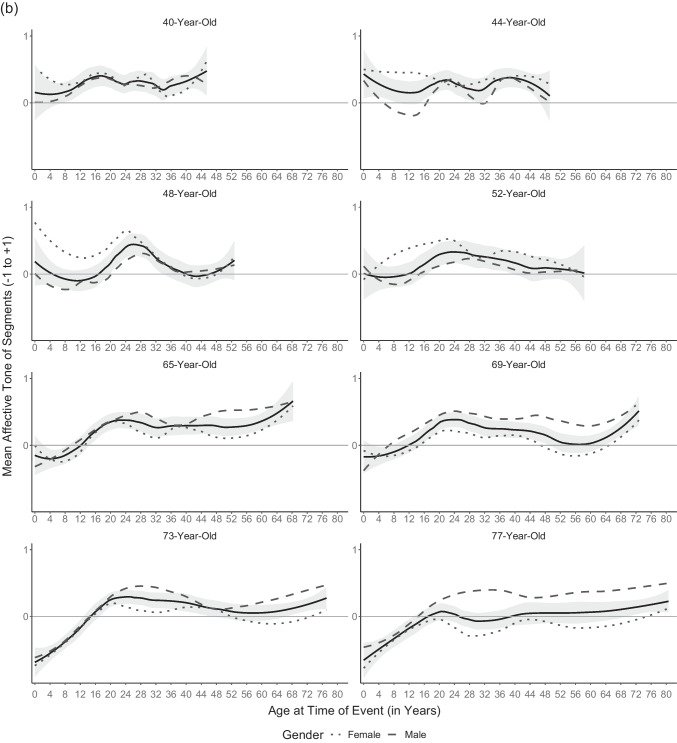
Mean affective tone of segments across the remembered lifespan in (**a**) younger participants and (**b**) middle-aged and older participants. *Note.* Trend lines according to the age groups’ current age were included using the loess method (α = .5) as implemented in the R package ggplot2 (Wickham, 2016); 95% confidence intervals are displayed in grey. Age groups of younger participants vary in size because they comprise varying numbers of cohorts: *n*_8yrs_ = 27, *n*_12yrs_ = 54, *n*_16yrs_ = 80, *n*_20yrs_ = 105, *n*_24yrs_ = 97, *n*_28yrs_ = 66, *n*_32yrs_ = 43, *n*_36yrs_ = 22. Middle-aged and older participants: *n*_40yrs_ = 28, *n*_44yrs_ = 22, *n*_48yrs_ = 20, *n*_52yrs_ = 20, *n*_65yrs_ = 30, *n*_69yrs_ = 29, *n*_73yrs_ = 28, *n*_77yrs_ = 27

To explore how current age influences the affective tone of memories from different times of one’s life, we plotted mean affective tone of segments by age at event separately for all age groups (not cohorts; Fig. 4). Four aspects of the overall shape of the variation of affective tone across the remembered life emerge from the plots. First, at all ages, beginnings are hard: the first years of life start not so positively, but then memories brighten up until grade school. Second, participants between ages 12 and their mid-30s remembered a decline after grade school years. What might be termed an *early adolescence dip* reaches a nadir point sometime between remembered ages 12 and 16. This remembered relative low point in early adolescence, however, started to fade in participants in their 30s and older. Third, at about the same age a positive peak began to emerge in the remembered 20s, or emerging adulthood, corresponding to the more positive normative transitions making up most of the reminiscence bump of autobiographical memory (Berntsen & Rubin, 2002). At least some traces of this positivity bump can be seen in all older age groups, with some variation. Thus, unlike the early adolescence dip, the positivity bump appears not to vary with age once it has emerged. Fourth, only the oldest cohort, born between 1938 and 1948, consistently remembered a negative childhood. This can be plausibly attributed to historical experiences of WWII and the immediate postwar years. In addition, what appears to be a most recent upswing of affective tone is an artefact of the plot with fewer participants contributing to the oldest remembered ages.

To test these four apparent effects post hoc, we ran separate three-level models including only segments from the remembered age period of interest (e.g., age at event 15 to 30 for the positivity bump in Cohorts 5 and 6); all were confirmed post-hoc (for more details, see Tables S3 to S6). First, affective tone of memories from early childhood (0 to 8 years of age at event) linearly increased with age at event (γ = 0.04, 95% CI [0.02, 0.05]) for all participants. Second, the early adolescence dip (8 to 20 years of age at event) showed in younger (below age 40; γ_Age at Event =_ −0.07, 95% CI [−0.12, −0.02]; γ_Age at Event_^2^_=_ 0.003, 95% CI [<0.01, <0.01]), but not older participants whose affective tone of memories increased linearly (γ_Age at Event_ = 0.03, 95% CI [0.02, 0.04]) leading up to the positivity bump of early adulthood. Third, this positivity bump between 15 and 30 years of age at event was confirmed for the two oldest cohorts (γ_Age at Event =_ 0.15, 95% CI [0.03, 0.26]; γ_Age at Event_^2^_=_ −0.003, 95% CI [−0.01, −0.00]). Fourth, comparing the three most frequent content categories of childhood memories showed that most negative childhood memories of the oldest cohort were war-related (Table 4).

Finally, we exploratorily compared the variation of affective tone of entire life narratives across the lifespan (participants’ current age; Fig. 1) with the variation of affective tone of memories with age at event (Fig. 4, ignoring remembered ages 0 to 8). The early adolescence dip and the positivity bump can be seen in both distributions. This implies that the two phenomena color both the affective tone of the entire life narrative at the time as well as later memories from the respective time. Moreover, adulthood can be described as relatively constant/monotonic in both distributions. However, growing older does change the shape of the remembered life in one respect. In retrospect, the early adolescence dip vanished behind the emerging positivity bump of early adulthood.

## Discussion

The purpose of this study was to gain a better understanding of how the affective tone of individuals’ past life is influenced by their current age and by the age at event when narrating their entire lives. The PE did not generalize to entire life narratives, but remembering the 20s was relatively positive already starting in the early 20s and turning into an early adulthood positivity bump in middle-aged and older adults. Unexpectedly, older participants told more negative life narratives as did early adolescents. Also, except for mid-adulthood, women told more negative lives. Consequently, affective tone of life narrative memories was influenced both independently as well as jointly by the age when life events were remembered and by the age when life events had been experienced.

Contrary to our first hypothesis, we did not find a PE of aging for life narrative memories, even though we studied a wide age range, but rather a reverse, negative effect. The few previous studies of the format of AMs closest to life narratives, life story AMs (Jensen et al., 2020; Thomsen et al., 2017), had found no PE either. The comparison to studies of cued and self-relevant AMs most of which found a PE suggests that the PE in AM highly depends on the method with which memories are elicited. In their meta-analysis, Reed et al. (2014) found that the positivity effect was absent in studies that had constrained information processing. We assume that the constraint exerted on retrieval and selection of AMs by asking for entire life narratives provides less leeway for using affective tone as a criterion than cue words do, because events are selected by criteria of self-relevance and representing the entire life in contrast to a more associative, bottom-up search. Constructing self-relevant AMs requires using some representation of one’s life as a frame of reference, the life story schema (Bluck & Habermas, 2000) as the highest level of the autobiographical knowledge base (a top-down process; Conway, 2005; Habermas et al., 2015; Koppel & Berntsen, 2015). Asking to represent the entire life puts even stronger constraints on the selection of memories, such as covering, at least cursorily, the entire lifespan lived so far, ordering memories chronologically, and creating some causal-motivational and thematic coherence. These constraints and the high cognitive load during the process of telling one’s entire life might weaken or abolish any PE. In addition, narrating how one has become the person one is today requires including both positive and negative events in order to appear credible. Besides these reasons for the absence of a PE in life narratives, the negative trend in older age also showed longitudinally, suggesting that negative experiences related to aging possibly contribute. We therefore suggest that the claim of a shift in preference from negative to positive stimuli with age in attention and in memory (Carstensen & DeLiema, 2018) needs to be updated for the memory part: The PE regards mainly memories triggered by external stimuli, but less effortful remembering of significant life memories and not at all of the entire life story.

As expected by the second hypothesis, we confirmed a positivity bump in the distribution of affective tone by age at event in entire life narratives, in the entire sample and particularly in the middle-aged and older group. Because a bump in a distribution requires lower values at both sides of it, the positivity bump begins to show only when affective tone in the 30s. Our findings showed that beginning in their early 20s young adults start to remember their 20s as more positive, which is in line with the few previous studies in which young adulthood was remembered more positively already by younger participants (Assink & Schroots, 2010; Berntsen & Rubin, 2002; Janssen & Murre, 2008; Thomsen et al., 2014). Thus, the remembered positivity of young adulthood could also be termed the *golden 20s*.

The positivity bump has been interpreted as reflecting the process of establishing an adult identity in emerging adulthood by mastering a series of culturally prescribed, normative status transitions. These culturally normative events are self-defining, especially memorable, and positive. Ample evidence for the oversampling of these positively evaluated normative events has been collected mainly in studies of the cultural life script, a set of most important events in a typical life (Berntsen & Rubin, 2004). The present study extends earlier cross-sectional findings on the positivity bump in three ways. First, whereas most studies have analyzed frequency distributions of positive and negative AMs separately, we demonstrated the positivity bump by plotting the mean affective tone which peaks in emerging adulthood. Second, the relative positivity is already present in young adults’ memories from emerging adulthood. Third, the 20s are golden in entire life narratives in two ways: Young adults tell more positive life stories than older adults, and they already remember this part of life more positively than earlier parts.

Data exploration provided four additional observations. First, women told more negative life narratives than men (except for Cohort 5). This is in line with some earlier findings such as Assink and Schroots’s (2002) study, with lower affective tone of life story AMs in women (Jensen et al., 2020), and also with the retrieval of fewer negative memories by older men (Ros & Latorre, 2010). From early years on, parents talk to their daughters more about emotions and particularly about sadness (Fivush & Zaman, 2014), females report more sadness (Chaplin, 2015), include more emotions overall in narratives of AMs than men (Grysman & Hudson, 2013), and experience more negative emotions when telling a recent past event (Pasupathi, 2003). Also, women tend to narrate more communal themes (Kemper & Habermas, 2021) and might therefore tend to include more others in their life narratives, which in turn is related to more negative life narratives (Altunnar & Habermas, 2018). Furthermore, as early as preadolescence, depression rates increase much more in females than in males (Cole et al., 2002; Hankin et al., 1998), and they remain higher throughout adulthood (Kessler, 2006). In our sample the negative effect of living without a partner was not gender specific. Thus, more negative life narratives of younger and older women might reflect psychological gender differences. Importantly, however, the more negative life narratives of women may well reflect first of all that women are in fact faced with more challenges throughout their lives due to societal restrictions (e.g., lower wages, difficulties in pursuing careers, pressure to conform to the normative “female” life). Taken together, more negative life narratives of women may have resulted from the gendered socialization of narrating more negative emotions, from higher rates of depression, and from more challenging life experiences.

A second observation was that both age distributions exhibited an early adolescence dip, that is, more negative life narratives of adolescents aged 12 and 16 as well as more negative memories from this period of life up to young adulthood. To our knowledge this age effect has gone unnoticed to date, probably due to the coarse resolution of age scales used. It is in line with the more negative affective tone of life narratives in 13- and 14½-year-olds’ than in 10½-year-olds (Ramsgaard & Bohn, 2021) as well as with developmental challenges of this life phase. Early adolescence is characterized by heightened emotional instability (Borghuis et al., 2017) in the context of puberty and the protracted development of prefrontal top-down regions (for regulation and control) versus subcortical regions involved in emotional processes (Casey et al., 2010), by increasing conflicts with parents and the struggle to become more autonomous from them (Hadiwijaya et al., 2017). Overall well-being takes a dip in early adolescence, more so for girls than for boys (e.g., Casas & González-Carrasco, 2019). Thus, the storm and stress (Arnett, 1999; Hall, 1904) of early adolescence might affect both the encoding and the reconstruction of AMs (Conway, 2005). Interestingly, the early adolescence dip vanished as participants grew older, suggesting a true interaction of the influences of age at event with growing older. The golden 20s seemed to obscure the earlier tough phase of early adolescence. This was not due to the fading affect bias (Skowronski, 2011; Walker et al., 2003), because childhood memories were not affected. This interaction adds an interesting systematic effect of aging on forgetting a specific life phase. The disappearance of the retrospective early adolescence dip might be due to motivational factors favouring a positive self-view or to identity-related motives favouring memories that match the rememberer’s adult identity better than those memories of being an awkward and disoriented early adolescent. Future studies should clarify the extent to which heightened emotionality in puberty as well as self-serving forgetting play a role in the early adolescence dip.

A third observation was that across remembered childhood, affective tone increased. Content of early negative memories was typical for childhood, such as beginning daycare (younger participants), illnesses and accidents (middle-aged participants), or family situations (all participants) including separation of parents (younger participants). This pattern corresponds to the narrative pattern described by McAdams (2013) as redemptive, in which difficult beginnings of life are increasingly overcome.

A fourth observation was that the atypically low affective tone of remembered childhood of elderly Germans who were born during WWII (birth years 1938–1948) was due to the prevalence of memories of (post-)war times. This corresponds to similar findings (Bohn, 2010; Glück & Bluck, 2007) as well as to war-related events generally leaving powerful negative AMs (Bohn & Habermas, 2016; Brown et al., 2009).

## Limitations and future directions

Several limitations of the study must be acknowledged. First, despite the wide age range of our sample, it did not include very old participants (81+) for whom a PE in entire life narratives cannot be excluded. Second, we externally coded the valence of memories but did not ask participants to evaluate the affective tone of their life narrative memories themselves. Although previous studies of life story AMs that were self-rated on affective tone did not find a PE either (Jensen et al., 2020; Thomsen et al., 2017), external and self-ratings of affectivity should be compared in the same study. Third, an experimental approach could compare the influence of varying constraints in different elicitation methods on the selection and the affective tone of AMs. Fourth, our finding of the early adolescence dip in both current age and age at event as well as the joint influences of both age parameters are based on exploratory analyses and therefore need to be confirmed in future studies. In turn, the specific strengths of the present study are the developmental lifespan sample with more than two age groups, the longitudinal design, and the use of to date understudied life narratives.

## Conclusion

This study contributes to the understanding of the independent and joint role of current age and age at event for the affective tone of AMs. It is the first study to test the PE and to find gender differences in affective tone of entire life narratives as well as to find the early adolescence dip of affective tone concurrently and retrospectively in life narratives. Findings on the absence of the PE in life narratives encourage focusing comparatively on different retrieval conditions when studying the PE in AMs and to specify under which conditions a PE does and does not show when remembering the personal past. The findings on the early adolescence dip extend developmental findings on a dip in well-being in early to mid-adolescence, supporting the notion of a normative phase of storm and stress in adolescence. We extended findings on a positivity bump to also show by mean affective tone peaking in remembered early adulthood for all participants. Moreover, we also found that the shape of the affective curve of the remembered life did change with growing older—namely, the early adolescence dip vanished once the positivity bump emerged. This combined finding and the early adolescence dip encourage studying effects of age and of age at time of the remembered events jointly in more detail as well as using a more fine-grained scale of age at event.

## Supplementary Information

ESM 1 (DOCX 964 kb)

## Data Availability

The datasets generated and analyzed in the current study and the analysis code are available at the Open Science Framework repository (https://osf.io/uj7ac/?view_only=3a151699b3d14d3185d698cbfb6cef32). Research materials are available by emailing the corresponding author. This study’s design and its analysis were not preregistered.

## Footnotes

1 In a recent yet unpublished study of life narratives of *N* = 61 elderly adults (https://osf.io/kda7t/), we did calculate interrater reliability for age estimates of life narrative segments, and the intraclass correlations were above .95, supporting our contention that dating is quite unambiguous.

2 Scatterplots (Fig. S1 equivalent to Figs. 1 and 3) showed that redemption and contamination were both related in comparable fashion to current age and age at event.
