# Supplementary material for: Lifespan effects of current age and of age at the time of remembered events on the affective tone of life narrative memories: Early adolescence and older age are more negative
Source: Mem Cognit. 2023 Feb 22;51(6):1265–86. doi: 10.3758/s13421-023-01401-x (PMC10368566; doi:10.3758/s13421-023-01401-x)
Supplement: Supplementary file 1 — (DOCX 964 kb) [file 13421_2023_1401_MOESM1_ESM.docx]

# Supplemental Material

| **Table S1**  *Dropout Analyses: Means, Standard Deviations, and One-Way Analyses of Variance in Overall Affective Tone of Life Narratives by Younger and Older Participants who Participated vs. did not Participate at the Subsequent Measurement Point* | | | | |
| --- | --- | --- | --- | --- |
|  | Younger | | Older | |
|  | No Dropout | Dropout | No Dropout | Dropout |
|  | *M*(*SD*) | *M*(*SD*) | *M*(*SD*) | *M*(*SD*) |
| Overall Affective Tone 2003 | 25.68 (24.72) | 20.39 (32.86) | — | — |
|  | *F*(1, 112) = 0.39, η² <.01 | | — | |
| Overall Affective Tone 2007 | 25.83 (24.83) | 24.16 (32.10) | 24.24 (21.88) | 8.23 (26.18) |
|  | *F*(1, 102) = 0.04, η² <.01 | | *F*(1, 56) = 3.15, η² = .05 | |
| Overall Affective Tone 2011 | 24.12 (25.44) | 31.34 (29.78) | 24.61 (24.94) | 3.40 (28.22) |
|  | *F*(1, 97) = 1.07, η² = .01 | | *F*(1, 49) = 1.93, η² = .04 | |
| Overall Affective Tone 2015 | 24.00 (22.13) | 19.09 (11.84) | 5.73 (26.89) | 34.73 (26.31) |
|  | *F*(1, 85) = 0.19, η² < .01 | | *F*(1, 46) = 2.23, η² = .05 | |
| *Note.* Mean overall affective (−100 to +100) tone was compared between participants who participated vs. did not participate four years later. Younger = Cohorts 1 to 4; Older = Cohorts 5 and 6. | | | | |

**Figure S1**

*Check Exclusion of Segments Coded for Redemption (a) and Contamination (b): Proportion in Entire Life Narratives by Participants’ by Current Age (Top) and Distribution of Segments Across the Remembered Lifespan Across all Participants by Age at Event (Bottom)*


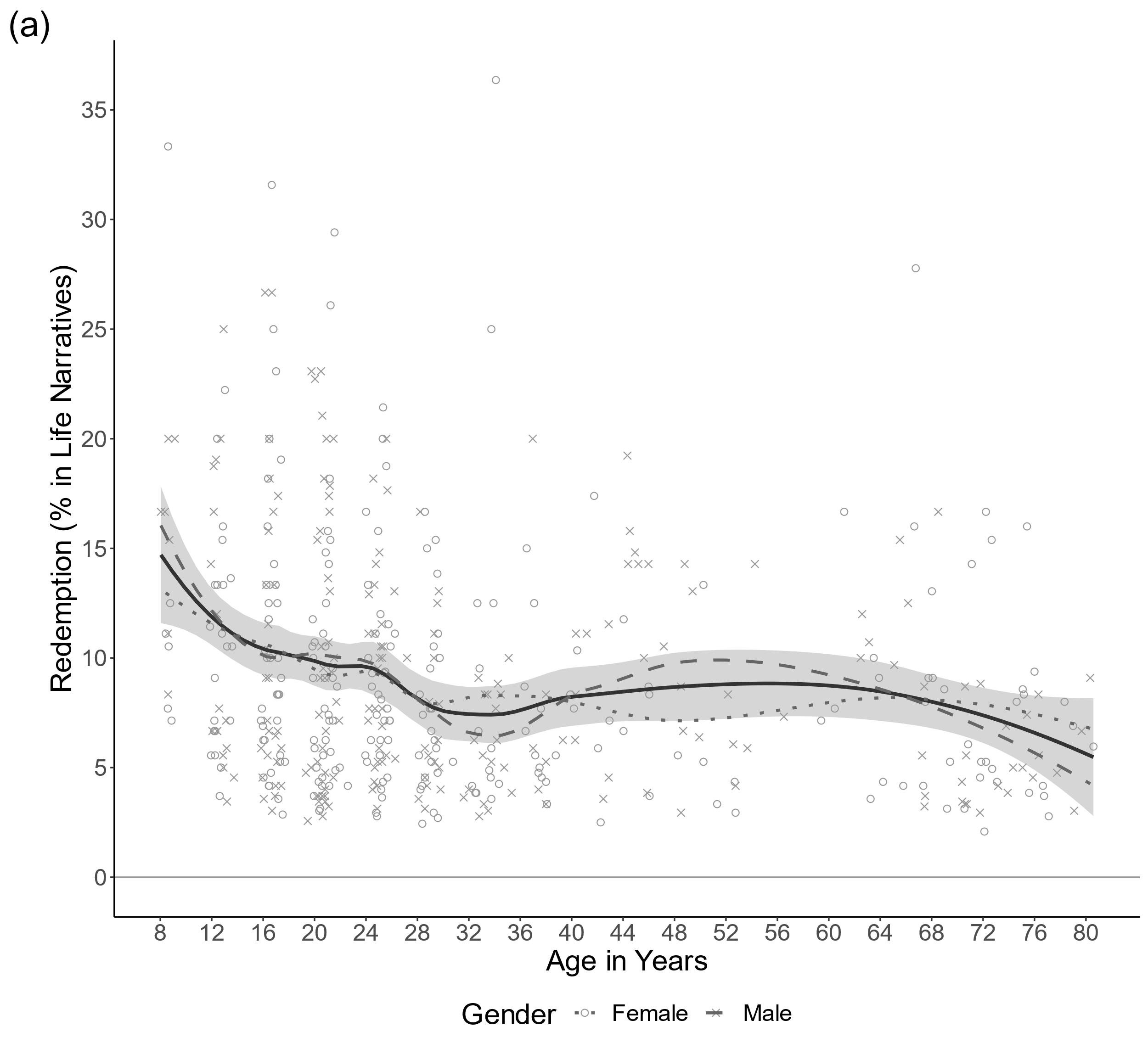

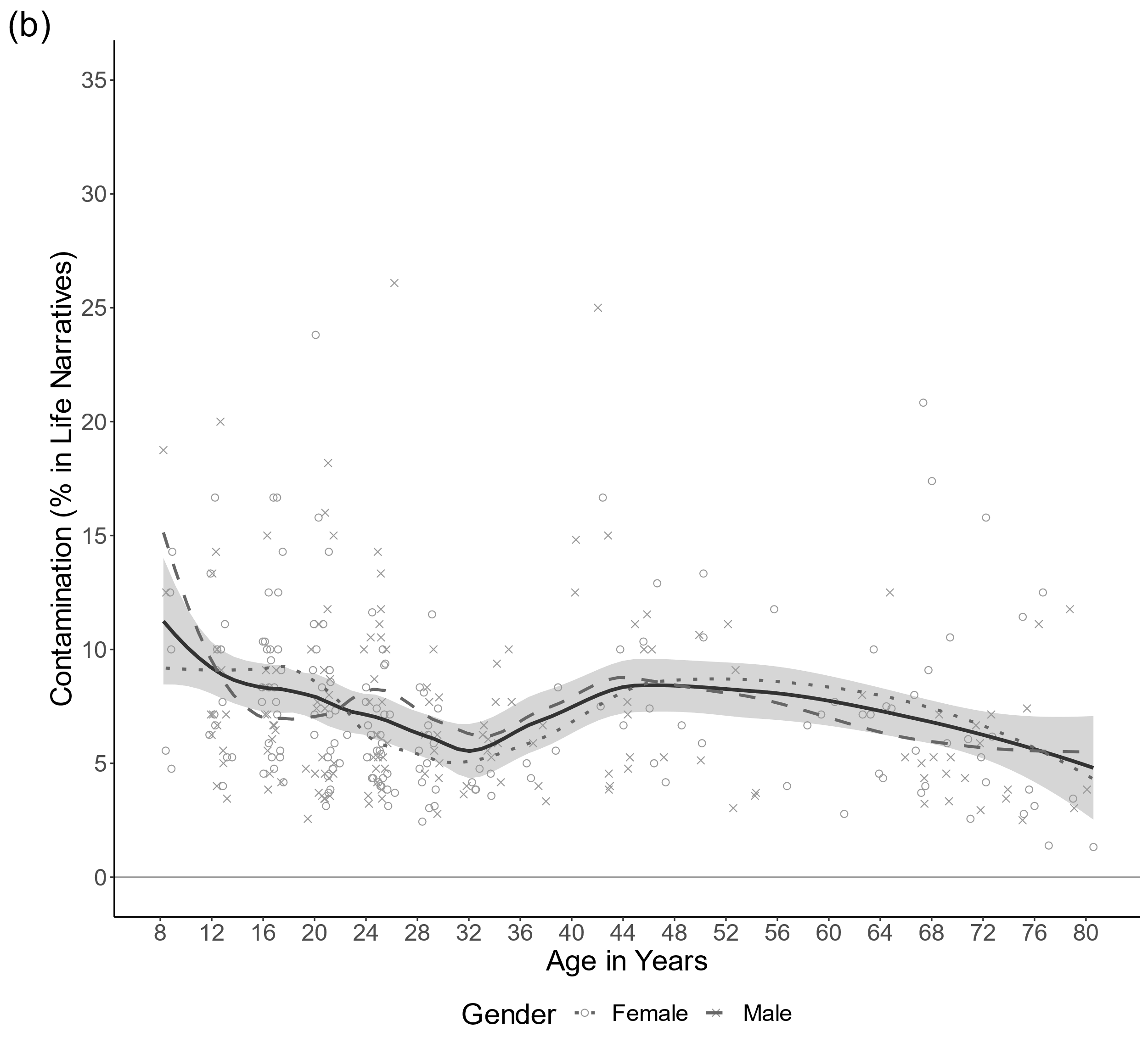


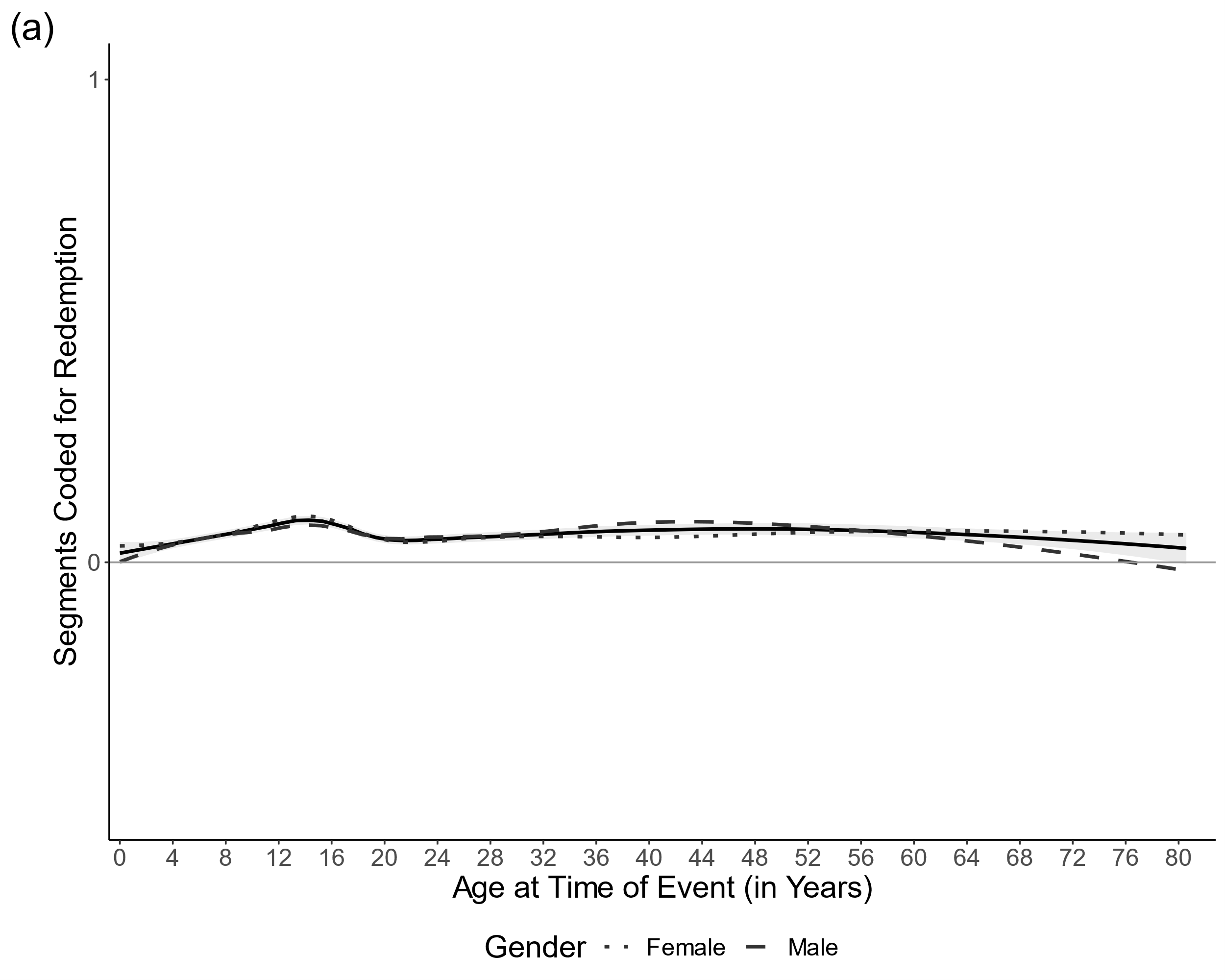

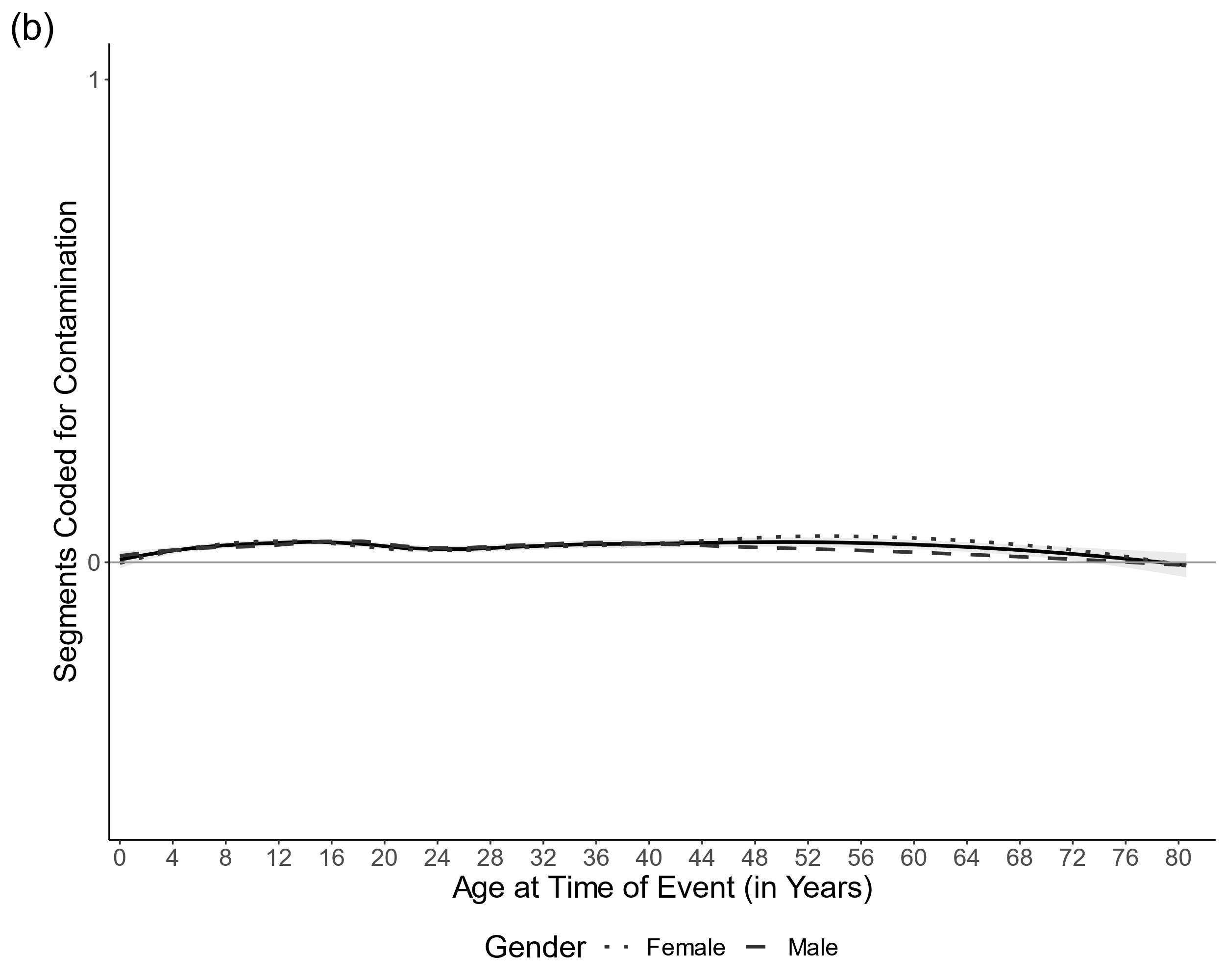


*Note.* Trend lines were included using the loess method (α = .5) as implemented in the R package ggplot2 (Wickham, 2016). The solid trend lines correspond to the overall mean. Top: Each point represents a life narrative. The 95% confidence interval of the overall mean is displayed in grey.

| **Table S2**  *Mean Overall Affective Tone by Age Group* | | | |
| --- | --- | --- | --- |
| Age group | *n* | *M* | *SD* |
| 8-Year-Old | 27 | 32.00 | 28.19 |
| 12-Year-Old | 54 | 21.43 | 28.14 |
| 16-Year-Old | 80 | 18.92 | 25.71 |
| 20-Year-Old | 105 | 26.20 | 24.21 |
| 24-Year-Old | 97 | 27.45 | 22.16 |
| 28-Year-Old | 66 | 28.93 | 20.65 |
| 32-Year-Old | 43 | 27.49 | 24.19 |
| 36-Year-Old | 22 | 21.93 | 25.49 |
| 40-Year-Old | 28 | 25.83 | 26.33 |
| 44-Year-Old | 22 | 25.01 | 24.33 |
| 48-Year-Old | 20 | 10.05 | 29.50 |
| 52-Year-Old | 20 | 16.58 | 26.54 |
| 65-Year-Old | 30 | 19.03 | 18.79 |
| 69-Year-Old | 29 | 16.53 | 25.98 |
| 73-Year-Old | 28 | 4.72 | 25.81 |
| 77-Year-Old | 27 | 4.35 | 29.84 |
| *Note*. Age groups of 12 to 32-year-old participants vary in size because they comprise varying numbers of cohorts. | | | |

| **Table S3**  *Multilevel Models Analysing the Increase in Affective Tone of Segments From Early Childhood (0 to 8 Years) of all Participants* | | | | | | |
| --- | --- | --- | --- | --- | --- | --- |
|  | Model 0 | | Model 1 | | **Model 2** | |
|  | Estimate (*SE*) | 95% CI | Estimate (*SE*) | 95% CI | Estimate (*SE*) | 95% CI |
| Fixed Part |  |  |  |  |  |  |
| Intercept | 0.17* (0.03) | [0.11, 0.23] | 0.16* (0.04) | [0.08, 0.24] | **−0.01 (0.05)** | **[−0.11, 0.09]** |
| Male |  |  | 0.01 (0.06) | [−0.11, 0.13] | **0.01 (0.06)** | **[−0.11, 0.13]** |
| Age at Event |  |  |  |  | **0.04* (0.01)** | **[0.02, 0.05]** |
| Random Variances |  |  |  |  |  |  |
| Level 1 $\sigma_{e}^{2}$ | 0.43 (0.66) | [0.64, 0.68] | 0.43 (0.66) | [0.64, 0.68] | **0.43 (0.66)** | **[0.64, 0.68]** |
| Level 2 $\tau_{2}^{2}$ | 0.02 (0.13) | [0.05, 0.19] | 0.02 (0.13) | [0.05, 0.19] | **0.02 (0.13)** | **[0.05, 0.19]** |
| Level 3 $\tau_{3}^{2}$ | 0.10 (0.32) | [0.28, 0.38] | 0.10 (0.32) | [0.27, 0.38] | **0.10 (0.32)** | **[0.27, 0.38]** |
| Model Fit |  |  |  |  |  |  |
| Deviance | 4777.17 | | 4777.12 | | **4744.02** | |
| Δ*χ*² |  |  | 0.04 | | **33.10*** | |
| df |  |  | 1 | | **1** | |
| *Note*. CI = confidence interval (based on profile method because bootstrapping led to non-convergence). *n* = 171 participants, *n* = 683 life narratives, *n* = 2239 segments. Variances are between segments ($\sigma_{e}^{2}),$ life narratives ($\tau_{2}^{2}),$ and participants ($\tau_{3}^{2})$. Sequentially adding predictors was tested with Δ*χ*^2^ tests based on model deviance (−2Log-Likelihood). Best model is in bold.  **p* < .05, one-tailed. | | | | | | |

| **Table S4**  *Multilevel Models Analysing the Early Adolescence Dip in Affective Tone of Segments (8 to 20 Years) of Younger Participants (Cohorts 1 to 4)* | | | | | | | | | | |
| --- | --- | --- | --- | --- | --- | --- | --- | --- | --- | --- |
|  | Model 0 | | Model 1 | | Model 2 | | **Model 3** | | Model 4 | |
|  | Estimate (*SE*) | 95% CI | Estimate (*SE*) | 95% CI | Estimate (*SE*) | 95% CI | Estimate (*SE*) | 95% CI | Estimate (*SE*) | 95% CI |
| Fixed Part |  |  |  |  |  |  |  |  |  |  |
| Intercept | 0.24* (0.02) | [0.20, 0.28] | 0.20* (0.03) | [0.15, 0.26] | 0.10* (0.05) | [−0.01, 0.21] | **0.61* (0.17)** | **[0.25, 0.96]** | 1.50* (0.03) | [−0.02, 2.90] |
| Male |  |  | 0.08* (0.04) | [−0.00, 0.16] | 0.07* (0.04) | [−0.00, 0.16] | **0.07* (0.04)** | **[−0.01, 0.16]** | 0.07* (0.04) | [−0.01, 0.16] |
| Age at Event |  |  |  |  | 0.01* (<0.01) | [<0.01, 0.01] | **−0.07* (0.02)** | **[−0.12, −0.02]** | −0.28* (0.16) | [−0.61, 0.08] |
| Age at Event² |  |  |  |  |  |  | **<0.01* (<0.01)** | **[<0.01, <0.01]** | −0.02 (0.01) | [−0.01, 0.04] |
| Age at Event³ |  |  |  |  |  |  |  |  | −0.00 (<0.01) | [−0.00, <0.01] |
| Random Variances |  |  |  |  |  |  |  |  |  |  |
| Level 1 $\sigma_{e}^{2}$ | 0.47 (0.69) | [0.67, 0.71] | 0.47 (0.69) | [0.67, 0.70] | 0.47 (0.69) | [0.67, 0.70] | **0.47 (0.69)** | **[0.68, 0.70]** | 0.47 (0.69) | [0.67, 0.70] |
| Level 2 $\tau_{2}^{2}$ | 0.03 (0.17) | [0.13, 0.20] | 0.03 (0.17) | [0.13, 0.20] | 0.03 (0.17) | [0.13, 0.20] | **0.03 (0.16)** | **[0.12, 0.19]** | 0.03 (0.16) | [0.13, 0.20] |
| Level 3 $\tau_{3}^{2}$ | 0.03 (0.18) | [0.14, 0.21] | 0.03 (0.17) | [0.13, 0.21] | 0.03 (0.17) | [0.13, 0.21] | **0.03 (0.17)** | **[0.13, 0.21]** | 0.03 (0.17) | [0.13, 0.21] |
| Model Fit |  |  |  |  |  |  |  |  |  |  |
| Deviance | 10748.02 | | 10744.66 | | 10739.10 | | **10729.76** | | 10728.13 | |
| Δ*χ*² |  |  | 3.36* | | 5.56* | | **9.34*** | | 1.63 | |
| df |  |  | 1 | | 1 | | **1** | | 1 | |
| *Note*. CI = confidence interval (based on bootstrapping). <0.01 = values between 0 and 0.01. *n* = 114 participants, *n* = 486 life narratives, *n* = 4981 segments.  Variances are between segments ($\sigma_{e}^{2}),$ life narratives ($\tau_{2}^{2}),$ and participants ($\tau_{3}^{2})$. Sequentially adding predictors was tested with Δ*χ*^2^ tests based on model deviance (−2Log‑Likelihood) from the previous model, except Model 5 was compared to Model 3. Best model is in bold.  **p* < .05, one-tailed. | | | | | | | | | | |

| **Table S5**  *Multilevel Models Analysing the Early Adolescence Dip in Affective Tone of Segments (8 to 20 Years) of Older Participants (Cohorts 5 and 6)* | | | | | | | | | | |
| --- | --- | --- | --- | --- | --- | --- | --- | --- | --- | --- |
|  | Model 0 | | Model 1 | | **Model 2** | | Model 3 | | Model 4 | |
|  | Estimate (*SE*) | 95% CI | Estimate (*SE*) | 95% CI | Estimate (*SE*) | 95% CI | Estimate (*SE*) | 95% CI | Estimate (*SE*) | 95% CI |
| Fixed Part |  |  |  |  |  |  |  |  |  |  |
| Intercept | 0.12* (0.04) | [0.04, 0.20] | 0.15* (0.06) | [0.04, 0.27] | **−0.32* (0.11)** | **[−0.54, −0.09]** | 0.21 (0.41) | [−0.59, 1.02] | 0.40 (1.79) | [−3.12, 3.92] |
| Male |  |  | −0.07 (0.08) | [−0.23, 0.09] | **−0.04 (0.08)** | **[−0.20, 0.11]** | −0.05 (0.08) | [−0.20, 0.11] | −0.05 (0.08) | [−0.20, 0.11] |
| Age at Event |  |  |  |  | **0.03* (0.01)** | **[0.02, 0.04]** | −0.05 (0.06) | [−0.17, 0.07] | −0.09 (0.41) | [−0.89, 0.71] |
| Age at Event² |  |  |  |  |  |  | <0.01 (<0.01) | [−0.00, 0.01] | 0.01 (0.03) | [−0.05, 0.06] |
| Age at Event³ |  |  |  |  |  |  |  |  | −0.00 (<0.01) | [−0.00, 0.00] |
| Random Variances |  |  |  |  |  |  |  |  |  |  |
| Level 1 $\sigma_{e}^{2}$ | 0.51 (0.72) | [0.68, 0.75] | 0.51 (0.72) | [0.68, 0.75] | **0.50 (0.71)** | **[0.67, 0.75]** | 0.50 (0.71) | [0.67, 0.74] | 0.50 (0.71) | [0.67, 0.74] |
| Level 2 $\tau_{2}^{2}$ | 0.01 (0.08) | [0.00, 0.19] | 0.01 (0.08) | [0.00, 0.19] | **0.01 (0.08)** | **[0.00, 0.18]** | 0.01 (0.09) | [0.00, 0.18] | 0.01 (0.09) | [0.00, 0.18] |
| Level 3 $\tau_{3}^{2}$ | 0.05 (0.22) | [0.15, 0.30] | 0.05 (0.22) | [0.15, 0.30] | **0.05 (0.21)** | **[0.14, 0.30]** | 0.05 (0.22) | [0.14, 0.30] | 0.05 (0.22) | [0.14, 0.30] |
| Model Fit |  |  |  |  |  |  |  |  |  |  |
| Deviance | 1985.42 | | 1984.74 | | **1963.12** | | 1961.33 | | 1961.32 | |
| Δ*χ*² |  |  | 0.68 | | **21.62*** | | 1.79 | | 0.01 | |
| df |  |  | 1 | | **1** | | 1 | | 1 | |
| *Note*. CI = confidence interval (based on profile method because of non-convergence with bootstrapping). <0.01 = values between 0 and 0.01. *n* = 58 participants, *n* = 193 life narratives, *n* = 888 segments. Variances are between segments ($\sigma_{e}^{2}),$ life narratives ($\tau_{2}^{2}),$ and participants ($\tau_{3}^{2})$. Sequentially adding predictors was tested with Δ*χ*^2^ tests based on model deviance (−2Log-Likelihood) from the previous model, except Model 5 was compared to Model 2. Best model is in bold.  **p* < .05, one-tailed. | | | | | | | | | | |

| **Table S6**  *Multilevel Models Analysing the Positivity Bump of Segments (15 to 30 Years) of Older Participants (Cohorts 5 and 6)* | | | | | | | | |
| --- | --- | --- | --- | --- | --- | --- | --- | --- |
|  | Model 0 | | Model 1 | | Model 2 | | **Model 3** | |
|  | Estimate (*SE*) | 95% CI | Estimate (*SE*) | 95% CI | Estimate (*SE*) | 95% CI | Estimate (*SE*) | 95% CI |
| Fixed Part |  |  |  |  |  |  |  |  |
| Intercept | 0.28* (0.04) | [0.20, 0.35] | 0.25* (0.05) | [0.15, 0.36] | 0.12 (0.12) | [−0.12, 0.36] | **−1.43* (0.67)** | **[−2.74, −0.12]** |
| Male |  |  | 0.05 (0.07) | [−0.10, 0.19] | 0.05 (0.07) | [−0.10, 0.19] | **0.05 (0.07)** | **[−0.09, 0.19]** |
| Age at Event |  |  |  |  | 0.01 (<0.01) | [−0.00, 0.02] | **0.15* (0.06)** | **[0.03, 0.26]** |
| Age at Event² |  |  |  |  |  |  | **−0.003* (<0.01)** | **[−0.01, −0.00]** |
| Random Variances |  |  |  |  |  |  |  |  |
| Level 1 $\sigma_{e}^{2}$ | 0.46 (0.68) | [0.65, 0.70] | 0.46 (0.68) | [0.65, 0.70] | 0.46 (0.68) | [0.65, 0.70] | **0.46 (0.68)** | **[0.65, 0.70]** |
| Level 2 $\tau_{2}^{2}$ | <0.01 (0.02) | [0.00, 0.12] | <0.01 (0.02) | [0.00, 0.12] | <0.01 (0.02) | [0.00, 0.12] | **0.01 (0.04)** | **[0.00, 0.12]** |
| Level 3 $\tau_{3}^{2}$ | 0.05 (0.23) | [0.18, 0.30] | 0.05 (0.23) | [0.17, 0.30] | 0.05 (0.23) | [0.17, 0.30] | **0.05 (0.23)** | **[0.17, 0.29]** |
| Model Fit |  |  |  |  |  |  |  |  |
| Deviance | 2824.24 | | 2823.79 | | 2822.31 | | **2816.79** | |
| Δ*χ*² |  |  | 0.46 | | 1.48 | | **5.51*** | |
| df |  |  | 1 | | 1 | | **1** | |
| *Note*. CI = confidence interval (based on profile method because bootstrapping led to non-convergence). <0.01 = values between 0 and 0.01. *n* = 58 participants, *n* = 203 life narratives, *n* = 1337 segments. Variances are between segments ($\sigma_{e}^{2}),$ life narratives ($\tau_{2}^{2}),$ and participants ($\tau_{3}^{2})$. Sequentially adding predictors was tested with Δ*χ*^2^ tests based on model deviance (−2Log-Likelihood) from the previous model. Best model is in bold.  **p* < .05, one-tailed. | | | | | | | | |
